# Therapeutic Response of Meniere’s Disease Utilizing Sorbus domestica

**DOI:** 10.7759/cureus.53702

**Published:** 2024-02-06

**Authors:** Richard Williams

**Affiliations:** 1 Geriatrics, Edward Via College of Osteopathic Medicine, Blacksburg, USA

**Keywords:** sorbus domestica, gemmotherapy, tinnitus, vertigo, meniere’s disease

## Abstract

Introduction

Meniere’s disease (MD) is a chronic condition characterized by episodic attacks of vertigo, fluctuating hearing loss, and tinnitus. MD can impart a significant socioeconomic impact with associated progressive hearing loss. First-line therapies consist of diuretics and antihistamines, with second-line therapies including intratympanic steroids and pulse therapy. Third-line treatments include endolymphatic sac surgery (ELSS) followed by intratympanic gentamicin injection and/or vestibular neurectomy.

The gemmotherapy Sorbus domestica's inherent properties to regulate venous circulation and lymphatic drainage have been utilized in the European literature for the treatment of MD and the patients in this study.

Methods

Patients presenting for rehabilitation at Pulaski Health and Rehabilitation Facility with a history of vertigo were examined and, through history and specific exam, to define MD. This resulted in six patients whose symptoms and exam were consistent with MD and interfered with their therapeutic progression. These patients were offered and accepted treatment with Sorbus domestica for their MD.

Results

All patients responded with either resolution or significant improvement in their symptoms and hearing loss. Treatment also resulted in an improved and probably shortened rehabilitative course. All patients had no adverse reactions and were supplied with resources for continual treatment upon discharge.

Conclusion

Sorbus domestica is a safe and viable treatment option for MD. It has been useful, especially in treatment-resistant diseases, without side effects and can be utilized in initial cases with improvement or resolution of hearing loss.

## Introduction

Meniere’s disease (MD) is a chronic condition first described in 1861 by French physician Prosper Meniere [1]. It has an estimated prevalence of 200-500 per 100,000 and is characterized primarily by episodic attacks of vertigo, fluctuating hearing loss, and tinnitus [2]. The etiology appears to be related to the abnormal quantity or composition of fluid in the inner ear. In 1937, endolymphatic hydrops (EH) was discovered in human temporal bones, revealing a potential pathologic counterpart to the syndrome described by Prosper Meniere [3]. Diagnosis is made clinically with symptom presentation and physical exam. The American Academy of Otolaryngology-Head and Neck Surgery (AAO-HNS) criteria define "Possible MD" as episodic vertigo or fluctuation hearing loss, "Probably MD" as one attack of rotatory vertigo lasting at least 20 minutes with tinnitus and documented hearing loss, and "Definite MD" as two or more spontaneous episodes of vertigo lasting at least 20 minutes with tinnitus and documented hearing loss [4]. The diagnostic criteria were updated by Lopez-Escamez in 2015, adding audiometric documentation of hearing loss [5]. Gadolinium contrasted 3T MRI can be used to visualize EH [6]. Multiple treatment modalities are used for MD with varying effectiveness. The international consensus (ICON) currently recommends diuretics and betahistine (not currently FDA-approved in the USA) as first-line therapy. It is estimated that 80% of patients are in remission of vertigo after first-line therapy, with tinnitus and hearing loss persisting [7]. The second-line treatment consists of intratympanic injection of corticosteroids and pulse treatment. The third-line treatment recommendations consist of either intratympanic injection of gentamicin, vestibular neurectomy, or labyrinthectomy [7].

Gemmotherapy is a specific type of herbal therapy that uses the properties inherent to embryonic tissue in growing plants, that is, the buds and new shoots of trees and shrubs. It has been defined as a comprehensive energetic cell therapy that adds constituents to activate the herb’s natural attributes and adds drainage attributes to the original herb properties [8]. Sorbus domestica is a species of Sorbus native to Europe, northwest Africa, and southwest that is extracted and used to regulate venous circulation and lymphatic drainage [9-11]. The extract is known for its serum hyperviscosity reduction and compensatory hypocoagulant reaction, believed to help regulate reticulo-endothelial metabolism. It is especially potent for disorders of hyperviscosity, believed to be a core component of the pathophysiology of MD [10].

We examined six patients treated with refractory MD over two years with Sorbus domestica. Sorbus domestica is not a widely accepted treatment for MD but is utilized in Europe for venous and lymphatic disorders [11]. We present six cases of intractable MD successfully treated with Sorbus domestica. All six patients met the criteria for "Definite MD" as defined by AAO-HNS; however, hearing loss was not confirmed via audiometry but did resolve on physical exam with gross hearing examination and utilizing the Weber and Rinne tests [12].

## Materials and methods

Gemmotherapy is the use of growing embryonic vegetal tissues such as young shoots buds and roots, prepared from a water-alcohol-glycerin mix from known therapeutic herbs. Thus, providing embryonic constituents that add additional growth and reparative elements to the original herbs’ properties. By utilizing the growth and repair elements through constituents such as auxins and gibberellins and in addition to combining the properties of drainage of cellular waste, these extracts show enhanced therapeutic properties. They are like tinctures but the addition of embryonic constituents separates them from the biopharmaceutics classification system (BCS) of gemmotherapy.

Sorbus domestica, one of the gemmotherapy herbs, also known as Service or Rowan tree, is a member of the Rosaceae family. Sorbus domestica is native to Europe, northwest Africa, and southwest [9]. Sorbus domestica is prepared from the fresh buds in the spring by maceration; then constituents are exsiccated at 1050°C. The maceration is then extracted in a 90% ethyl alcohol and glycerol 1:1 mixture with the final weight 20 times the original weight of the material. The maceration is periodically mixed at room temperature for three weeks. The mixture is then decanted and filtrated at 107 Pascal and kept for 48 hours, then refiltered, and adjusted to a ratio of 1:20 resulting in the basic glyceric macerated (MG). The MG is then diluted to a ratio of 1:10 resulting in the final solution of 50:30:20 water:ethylalchohol:glycerol, resulting in 100 mL of 1DH corresponding to 0.50 g of the original desiccated product [11]. Specific constituents are phenolics, flavonoids, and tannins along with macro- and micronutrients. Gemmotherapy possesses high antioxidant activity [11]. Specific properties are equilibration of hyperviscosity, and anti-inflammatory of the veinous system with specific tropism of the medium and internal ear. It also possesses drainage activity of the lymphatic system with a high affinity for the central nervous and auditory systems [13]. Specific indications include hearing loss, MD, tinnitus, and otosclerosis associated with circulation dysfunction [14].

Six patients presenting for rehabilitation at Pulaski Health and Rehabilitation Facility Center, which usually admits more aged patients, with a history of vertigo were examined specifically to differentiate the diagnosis of vertigo, resulting in the diagnosis of MD for the patients who fulfilled the required criteria [4,5]. The physical exam utilized the Dix-Hallpike maneuver, gross hearing with finger rub test, and Rinne Weber test, along with visualization of the tympanic membrane [15-17]. Patients diagnosed with MD that interfered with their rehabilitation progression were offered treatment with Sorbus domestica. The patients were informed that this was not a standard or an approved regime for MD in the United States or Virginia, but there was literature for its utilization in MD in Europe, and it was an herbal therapy that had been and was continually used in the facility. Patients who agreed to its use were supplied the herb with 3 mL of 0.1 mg/mL solution of Sorbus domestica three times daily and re-evaluated at two days and one week. As all patients responded, the treatment was continued throughout their rehabilitation. After discharge, patients were given information about obtaining and continual use. All gemmotherapy was obtained from certified distributors, including Borion: United States Pharmacopeia (USP) certified, Marleen Herbs: Australian certified organic, Herbiolys: Certified organic agriculture, and HerbalGem: German Pharmacopoeia certified.

## Results

The initial patient was not a rehabilitation patient, but a 45-year-old female. She has continued the Sorbus for 13 years, has been well-controlled without hearing loss, and was able to reduce the medication to two droppers four times a week for maintenance. She was not included in the article because she was not a rehabilitative patient, but served as the initiating case for use in the nursing home. Her initial diagnosis was made by an ENT physician and confirmed at the University of Virginia ENT Department. For this article, six patients, four females and two males, with intractable MD were admitted to a rehabilitation facility secondary to various acute and chronic ailments unrelated to MD. Their ages ranged from 62 to 81, with a median age of 77. All six patients met the criteria for "Definite MD" as defined by AAO-HNS, with two or more spontaneous episodes of vertigo lasting at least 20 minutes with tinnitus and hearing loss, with all symptoms beginning a minimum of three months before admission. All patients had previously attempted diuretic therapy for MD or were on diuretics on admission, secondary to other comorbidities. No intratympanic injections were attempted. Prior to treatment, each patient reported multiple daily attacks of vertigo lasting greater than 20 minutes, tinnitus, and hearing loss consistent with symptoms they experienced prior to admission. They were treated with 3 mL of 0.1 mg/mL solution of Sorbus three times daily for one week and re-evaluated. All six patients reported symptomatic improvement in all aspects of their MD, including vertigo, tinnitus, and hearing loss. Four patients (three female and one male) reported complete resolution of their symptoms, while two reported significantly decreased symptom frequency. No side effects or complaints were reported at either interval. Previous use of diuretics resulted in minimal improvement in their MD and were continued for use in hypertension and swelling only (Table 1).

**Table 1:** Summation of six patients treated for MD with Sorbus domestica Six patients were treated, ages 62 to 81 with a median age of 77. All patients responded within three days with improvement in hearing and vertigo. Complete resolution of symptoms in four out of six patients with significant improvement in vertigo and hearing loss in all others. The two patients with partial response changed from vertigo to dizziness and had significant cerebral vascular disease as noted on the CT scan. One patient with dizziness improved with the addition of Gingko, which improves cerebral perfusion. Additional symptoms of lower extremity edema and restless legs also responded to Sorbus domestica treatment. Therapy was well-tolerated without side effects or associated complaints. HCTZ, hydrochlorothiazide; ASCVD, arteriosclerotic cardiovascular disease; N/A, not applicable; MD, Meniere's disease

Admitting diagnosis	Diuretics	Hearing return	Time to reduction of symptoms	Associated symptoms response	Improvement/duration of stay
Stroke	Furosemide	Yes	3 days	Decreased restless legs	Full/4 years
Hip fracture	HCTZ	Yes	2 days	N/A	Full/14 weeks
Thermal burns	Previous treatment	Yes	2 days	Restless legs	Moderate with significant ASCVD/4 months
Patellar fracture	Previous treatment	Yes	2 days	N/A	Full/4 weeks
Periprosthetic fracture hip joint	Spironolactone/furosemide	Yes	2 days	Lower extremity edema and restless legs	Full/4 weeks
Falls chronic anemia	Furosemide	Yes	2 days	Restless legs ASCVD	Moderate/14 weeks/ASCVD

## Discussion

All patients reported improvement of symptoms within three days and significantly decreased frequency after one week, with continued remission of MD until the end of their treatment stay. Notably, patients reported improvement or resolution in episodic vertigo, tinnitus, and hearing loss, which are typically more refractory to conventional therapies, including diuretics and intratympanic injections of steroids or gentamicin, which worsens hearing loss [18]. Interestingly, there was also improvement in restless legs and lower extremity edema in selected patients. According to a nationwide survey in 1977, an average of 54.7% of MD patients were unable to function normally in their daily lives. These patients mostly complained of repeated attacks of vertigo [19].

**Figure 1:**
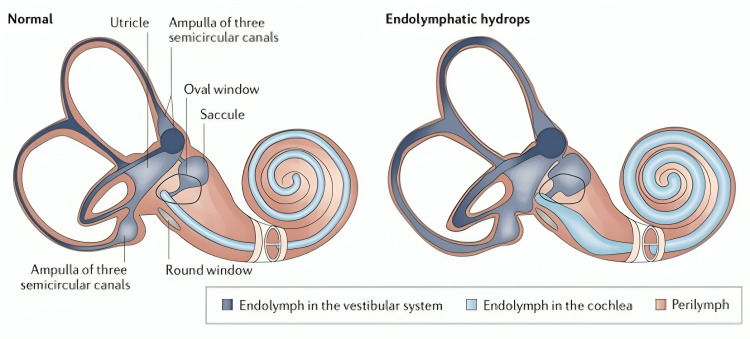
Examples of two labyrinths, one normal and the other with EH EH, endolymphatic hydrops De Luca P, Cassandro C, Ralli M, Gioacchini FM, Turchetta R, Orlando MP, Iaccarino I, Cavaliere M, Cassandro E, Scarpa A. Dietary Restriction for The Treatment of Meniere's Disease. Transl Med UniSa. 2020 May 31;22:5-9 PMC7265917. open-access article distributed under the terms of the Creative Commons Attribution License

EH is a pathological condition that results in the accumulation of fluid in the endolymphatic fluid representing a pathologic anatomic finding in which the structures bounding the endolymphatic space are distended by an enlargement of endolymphatic volume and is the defining pathological indicator of MD [20]. Although EH is seen in all patients diagnosed with MD, the reverse is not true; all patients with EH do not necessarily demonstrate symptoms of MD [21]. The findings by Merchant et al. question the established dictum that hydrops is the physiologic endpoint of MD symptoms [21]. Rather, the authors suggest that EH is a manifestation of labyrinthine homeostasis that can result in MD and can be caused by many different processes (Figure 1) [20,22].

Although there may be multiple causes of MD, if a cause is defined, such as head trauma or a genetic cause or migraines, then the symptoms are defined as Meniere’s syndrome, and treatment of the primary cause is recommended [23,24]. Treatment of MD usually starts with a conservative approach. Recent studies concluded that people with mild disease were more likely to respond to the conservative approach [25]. This approach consists of lifestyle changes, including imposing restrictions on caffeine, alcohol, and tobacco due to their vasoconstrictive and diuretic properties, which may exacerbate hydrops, as recommended by AAO-HNS [26].

Patients have described acute symptoms after high salt exposure, and treatment based on sodium restriction has been a foundation of the management of MD since the 1930s [27]. Sodium intake does have a myriad of effects on vascular volume, arterial stiffness, and physiologic changes that could be helpful in MD [28].

Plasma osmolality, rather than absolute sodium levels, has led some researchers to shift focus from salt/sodium toward the transport and regulation of water itself. Naganuma et al. found that increased water intake lowers ADH levels and more effectively improves and prevents HL compared with conventional therapy [29].

After lifestyle changes conservative medical treatment is recommended. This consists of betahistine and diuretics [7]. Other treatment modalities considered as conservative or complimentary include positive pulse therapy (Meniett or P-100 device) [30]. Osmotic diuretic use in MD with Isosorbide (90 mL/day) has been used as an alternative to other diuretics to prevent vertigo attack recurrence and to decrease the volume of endolymph without recurrence in rats [31]. It has also shown good outcomes in reducing symptoms and improving hearing clinically [32].

Intratympanic corticosteroids (ITC) are sometimes administered directly into the middle ear to treat this condition (through the tympanic membrane). The underlying cause of MD is unknown, as is the way in which this treatment may work. The efficacy of this intervention in preventing vertigo attacks and their associated symptoms is currently unclear but can be utilized although involves injection through the tympanic membrane and is considered a simple procedure [7].

**Figure 2:**
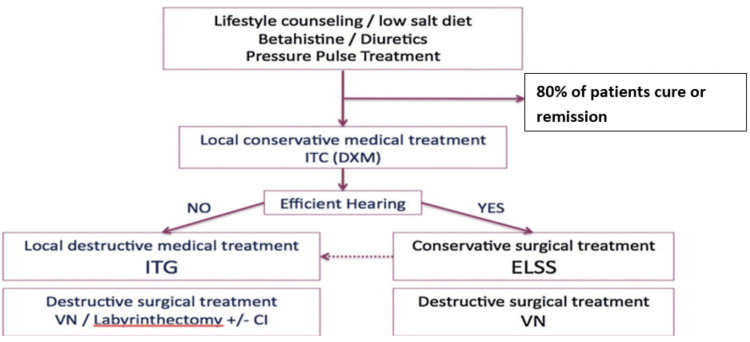
ICON algorithm for the treatment of MD The figure represents a proposal of an algorithm for MD treatment as an ICON obtains for the IFOS meeting 2017. The first line of treatment includes medical conservative treatment. After this line of treatment, 80% of patients with MD are cured or in remission. Then the second line is the IT injections, mainly ITC as a conservative treatment and ITG in case of failure and preferentially in patients with hearing impairment. After this second line, 90% to 95% of the patients are cured or in remission. The third line is the surgical, conservative, or destructive treatment. If indicated, ELSS must be indicated before ITG. ITC, intratympanic injection of corticosteroids; DXM, dexamethasone; ITG, intratympanic injection of gentamycin; ELSS, endolymphatic sac surgery; VN, vestibular neurectomy; labyrinthectomy +/- Cl (with and without gentamycin); ICON, international consensus; MD, Ménière's disease Reproduced from Nevoux J, Barbara M, Dornhoffer J, Gibson W, Kitahara T, Darrouzet V: International consensus (ICON) on treatment of Ménière’s disease. Eur Ann Otorhinolaryngol Head Neck Dis. 2018, 135:S29–32. 10.1016/j.anorl.2017.12.006 Permission by permissionfranceelsevior.com

Endolymphatic sac surgery (ELSS) should be considered as the next option after failure of conservative medical treatment, especially if hearing is preserved. In contrast to chemical labyrinthectomy and vestibular neurotomy, ELSS is considered a conservative surgical treatment (Figure 2) [7].

With the need for additional medicines, Sorbus may provide needed additional treatment for MD [19]. Sorbus domesticus could function as a viable alternative therapy to treat MD, with an excellent safety profile, no known drug interactions or side effects, and ease of administration. With the documented need for better and more effective treatments, this could provide a needed addition to the therapeutic regime and the ability to restore hearing loss and successful treatment of recalcitrant disease. We propose a follow-up with a randomized control trial with increased sample size and audiometric studies and potentially contrasted 3T MRI to gather more objective data.

Given Sorbus domestics’ success with other venous and lymphatic circulation disorders, the mechanism would appear to be related to reduced viscosity of the endolymphatic fluid in the inner ear and improved drainage of the inner ear by both venous and lymphatic mechanisms [13].

Limitations

The number of patients is small, utilizing a larger population may better define specific populations that would not respond to treatment or specific patients for whom the treatment would be most efficacious. Specific audiometric measuring was not performed and is part of the expanded criteria for Mineriere's disease. Further studies should include specific documented hearing changes. Gemmotherapy use and documentation need to be expanded to define its use in current treatment.

## Conclusions

MD can cause significant physical and social sequelae in affected patients. Although treatment is available, hearing loss and tinnitus can still progress after initial response to treatment. The results utilizing Sorbus domesticus showed improvement in all symptoms. These responses indicate that Sorbus domesticus could function as a viable alternative therapy to treat MD, with an excellent safety profile, no known drug interactions or side effects, and ease of administration. With the documented need for better and more effective treatments, this could provide a needed addition to the therapeutic regime. We propose follow-up with a randomized control trial with increased sample size and audiometry and potentially contrasted 3T MRI to gather more objective data.
